# Mitochondrial Dysfunction Involved in the Cytotoxicity of Tramadol in Human Endometrial Carcinoma Cells

**DOI:** 10.3390/ijms24010099

**Published:** 2022-12-21

**Authors:** Li-Chun Liu, Zih-Syuan Wu, Jia-Lin Chen, Zhi-Fu Wu, Hou-Chuan Lai, Yi-Hsuan Huang

**Affiliations:** 1 Department of Obstetrics and Gynecology, Tri-Service General Hospital, National Defense Medical Center, Taipei City 114, Taiwan; 2 Division of Obstetrics and Gynecology, Tri-Service General Hospital Songshan Branch, National Defense Medical Center, Taipei City 105, Taiwan; 3 Graduate Institute of Life Sciences, National Defense Medical Center, Taipei City 114, Taiwan; 4 Department of Anesthesiology, Tri-Service General Hospital, National Defense Medical Center, Taipei City 114, Taiwan; 5 Graduate Institute of Medical Sciences, National Defense Medical Center, Taipei City 114, Taiwan; 6 Department of Anesthesiology, Kaohsiung Medical University Hospital, Kaohsiung Medical University, Kaohsiung City 807, Taiwan; 7 Department of Anesthesiology, Faculty of Medicine, College of Medicine, Kaohsiung Medical University, Kaohsiung City 807, Taiwan; 8 Center for Regional Anesthesia and Pain Medicine, Wan Fang Hospital, Taipei Medical University, Taipei City 116, Taiwan

**Keywords:** tramadol, endometrial cancer, mitochondrion, reactive oxygen species, adjuvant therapy

## Abstract

Tramadol is a common anesthetic used to treat cancer pain, including endometrial cancer, but its function in endometrial cancer remains unclear. The purpose of this study was to elucidate the antitumor effects of tramadol on human endometrial cancer cells. Colony formation, BrdU, cell cycle profiles, apoptosis, ROS, and Western blot analyses were used to study the response of endometrial cancer cells to tramadol. JC-1 and seahorse metabolic flux assays were used to detect the effect of tramadol on mitochondria in endometrial cancer cells. Combination index was used to detect the interaction of tramadol with chemotherapy drugs in endometrial cancer cells. In this study, we found that tramadol was able to inhibit proliferation and induce cell cycle arrest, ROS generation, and apoptosis in two types of endometrial cancer cells. In addition, tramadol treatment also induced mitochondrial dysfunction in endometrial cancer cells by causing a loss of mitochondrial membrane potential and a decreased oxygen consumption rate. More importantly, the synergetic effect of tramadol with doxorubicin or cisplatin was further confirmed in endometrial cancer cells by the results of the combination index and apoptosis assay. In summary, our findings indicate that tramadol has an antitumor effect on endometrial cancer cells, which might serve as a potential adjuvant therapy strategy for endometrial cancer.

## 1. Introduction

Endometrial cancer is one of the most important cancers in women, and it has a growing trend of incidence. There were approximately 410,000 new cases worldwide in 2020 [1,2]. Due to easily found abnormal vaginal bleeding, endometrial cancer is often diagnosed at an early stage. Surgeries including hysterectomy are initially the most common standard management. Adjuvant chemotherapy is recommended for patients with the presence of poor prognostic features, such as a high-grade histologic type and deep myometrium invasion [3]. Despite most endometrial cancers being treated early, 5-year disease-free survival for patients with pelvic or para-aortic lymph node metastases is around 30–70% [4], and salvage chemotherapy is advised for cancer relapses. Therefore, improving disease-free survival and general outcome and decreasing potential chemotherapy side effects for endometrial cancer patients are crucial.

Endometrial cancers are divided into two subtypes based on histology [5]. Type I, the most common subtype, consists of endometrioid adenocarcinomas. Type II is composed of several non-endometrioid adenocarcinomas, such as serous or clear-cell carcinoma. Most endometrial cancers are related to a genetic problem. The endometrioid and clear-cell types are commonly associated with phosphatase and tensin homolog (PTEN) mutation or loss, while serous and carcinosarcoma types are frequently related to the p53 mutation [6]. In type I endometrial carcinoma, the p53 mutation frequency is approximately 10–40%, and it is approximately 90% in type II endometrial carcinoma [7]. P53 mutations are predictive of prognosis in endometrial cancer and are associated with unfavorable outcomes. PTEN is a tumor-suppressor gene that plays a key role in negatively regulating the PI3K/AKT signaling pathway, and also regulates various biological processes such as apoptosis, metabolism, and growth [8]. Considering the genetic mutations and signaling pathways involved in the pathogenesis of endometrial cancer, type I is usually low-grade and estrogen-dependent, and often has an excellent prognosis, while type II is not. It is necessary to develop a compelling therapeutic strategy for endometrial cancer, especially for type II patients.

Tramadol is a kind of opioid analgesic that is commonly used for the control of postoperative pain, chronic pain, and cancer pain. It is a centrally acting analgesic with weak affinity for opioid receptors and affects the reuptake of norepinephrine and serotonin in the synapses [9]. Recently, studies have shown that tramadol has anti-tumor effects in different cancers, such as breast cancer and lung cancer [10,11]. These effects are involved in the epithelial–mesenchymal transition (EMT), migration, invasion, colony formation, and signaling pathways [12]. Our previous work demonstrated a novel therapeutic strategy for the combination of tramadol and doxorubicin in breast cancers [11]. In addition, an in vitro validated retrospective clinical study showed that breast cancer patients treated with tramadol after surgery had a 0.71-fold lower risk of recurrence and a 0.56-fold lower mortality rate [10]. However, whether tramadol has a cytotoxic effect on endometrial cancer remains unclear.

Mitochondria are the energy factories of cells. A cell’s primary energy source is the use of oxygen and simple sugars to produce adenosine triphosphate (ATP) through the process of oxidative phosphorylation (OXPHOS) that occurs on the mitochondrial electron transport chain (ETC) [13]. However, the ETC process is not perfect. The production of mitochondrial reactive oxygen species (mtROS) mainly occurs during the OXPHOS process, where electron leakage in complexes I and III leads to partial reduction of oxygen to form superoxide (O_2_.^−^). Cellular homeostasis is tightly controlled by the regulation of cell death and cell survival processes such as apoptosis and autophagy through mitochondria and reactive oxygen species (ROS) signaling [14]. Cancer initiation and progression is often associated with a slight increase in ROS [15], but high levels of ROS can induce cell death by activating several signaling pathways that lead to apoptosis [16]. Therefore, in addition to the role of mitochondria in energy metabolism, the regulation of cell death is also a major function of these organelles. Targeting the increase in ROS levels in cancer cells beyond a threshold could be a potential strategy for cancer therapy.

Due to the differential responses of chemotherapy in type I and type II endometrial cancers [17], we first sought to determine whether tramadol had an antitumor effect on RL95-2 and HEC-1-A cells. We further assessed whether this was synergistic with current chemotherapeutic drugs, including doxorubicin and cisplatin, in endometrial cancer. Repurposing tramadol might provide new insights into current chemotherapy for endometrial cancer to overcome drug resistance or to reduce chemotherapy doses and the potential side effects.

## 2. Results

### 2.1. Tramadol Suppressed the Proliferation of Human Endometrial Cancer Cells

**Figure 1:**
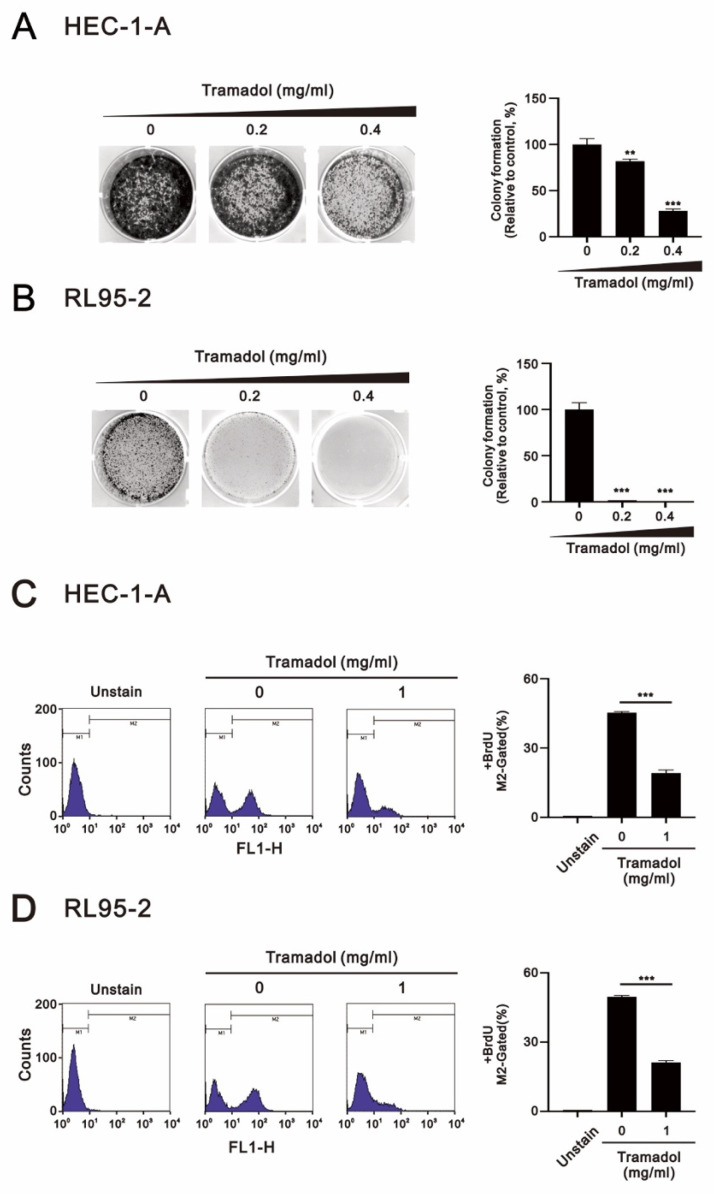
Effects of tramadol on proliferation in human endometrial cancer cells. (**A**,**B**) HEC-1-A and RL95-2 cells were treated with tramadol (0, 0.2, and 0.4 mg/mL) for 14 days. (**C**,**D**) HEC-1-A and RL95-2 cells were treated with tramadol (0 and 1 mg/mL) for 24 h. Cell proliferation was assessed using BrdU assay with flow cytometry. M2-gated shows the proportion of proliferating cells. Bars depict the mean ± SD of three independent experiments. Student’s *t*-tests were performed and the results were compared with the vehicle. ** *p* < 0.01, and *** *p* < 0.001 (Student’s *t*-test).

The ability of cancer cells to escape growth inhibition is a hallmark ability that is highly complementary to sustaining proliferative signaling. Mutations in multiple genes encoding tumor suppressor proteins that inhibit cell growth and proliferation have been identified in endometrial cancer cells, such as TP53 (deletion) and PTEN (missense mutation) in RL95-2 cells and TP53 (missense mutation) and KRAS (missense mutation) in HEC-1-A cell [18]. Therefore, to determine whether tramadol affects the survival and proliferation of endometrial cancer cells, we examined the response of both HEC-1-A and RL95-2 cells to tramadol in colony formation assay, the vehicle of tramadol was double-distilled water. Figure 1A,B show that tramadol significantly inhibited cell proliferation and survival of HEC-1-A and RL95-2 cells. However, RL95-2 was more sensitive to tramadol than HEC-1-A cells.

Since measuring DNA synthesis is one of the most accurate ways to detect cell proliferation, we combined flow cytometry with BrdU, which is incorporated into DNA during the S phase of the cell cycle, to determine whether tramadol inhibits the proliferative capacity of HEC-1-A and RL95-2 cells. As shown in Figure 1C,D, we found that tramadol significantly reduced the proliferative capacity of HEC-1-A and RL95-2 cells.

**Figure 2:**
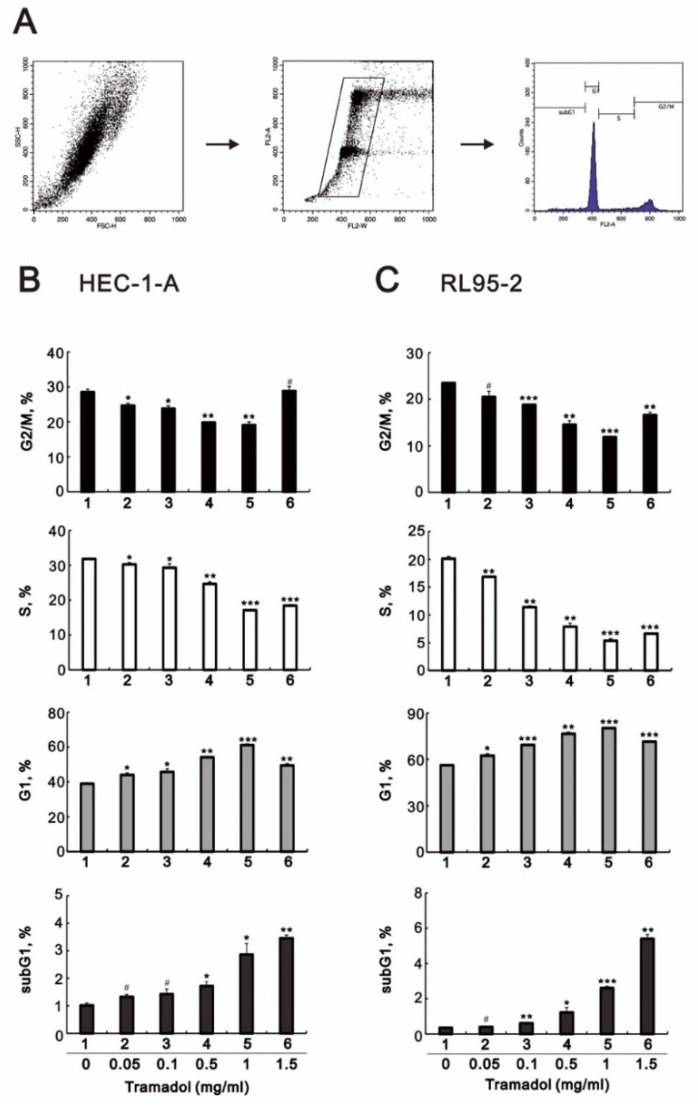
Effects of tramadol on the cell cycle of human endometrial cancer cells. (**A**) Representative images of individualized gating strategies for different cell cycle phases using PI staining (**B**,**C**) HEC-1-A and RL95-2 cells were treated with tramadol (0, 0.05, 0.1, 0.5, 1, and 1.5 mg/mL) for 24 h. Cells were stained with propidium iodide (PI) and analyzed using flow cytometry. Bars depict the mean ± SD of three independent experiments. Student’s *t*-tests were performed and the results were compared with the vehicle. ^#^ *p* > 0.05, * *p* < 0.05, ** *p* < 0.01, and *** *p* < 0.001 (Student’s *t*-test).

We then assessed the effect of tramadol on cell cycle progression in HEC-1-A and RL95-2 cells using flow cytometry. The gating strategy shown in Figure 2A was used. As shown in Figure 2B,C, we found that tramadol caused a significant accumulation of cells in the G1 and sub-G1 phases and a decrease in the proportion of cells in the G2/M and S phase in HEC-1-A and RL95-2 cells. The decrease in the proportion of cells in the S phase is consistent with our previous cellular proliferation results shown Figure 1C,D. We also found a significant enhancement in cell proportion in the sub-G1 phase, indicating that tramadol induced DNA fragmentation in HEC-1-A and RL95-2 cells, which occurred at the later stage of apoptosis.

### 2.2. Tramadol Caused Different Types of Cell Death in Two Types of Human Endometrial Cancer Cells

**Figure 3:**
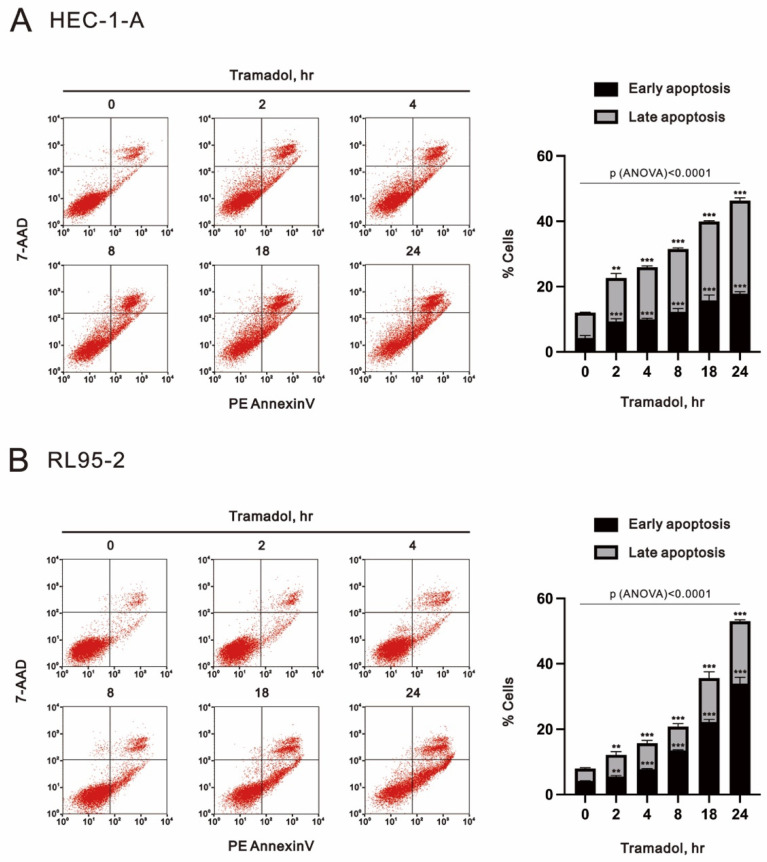
Time-course effects of tramadol on apoptosis in human endometrial carcinoma cells. (**A**,**B**) HEC-1-A and RL95-2 cells were treated with 1 mg/mL tramadol for 0, 2, 4, 8, 18, and 24 h. Black bars represent the proportion of early apoptotic cells, and gray bars represent the proportion of late apoptotic cells. Bars depict the mean ± SD of three independent experiments. Student’s *t*-tests were performed, and the results were compared with the vehicle. ** *p* < 0.01, and *** *p* < 0.001 (Student’s *t*-test). (**A**,**B**) The means between the groups were analyzed by one-way ANOVA.

Next, we assessed the apoptosis of endometrial cancer cells that were exposed to time-course or increasing concentrations of tramadol using flow cytometry with annexin V and 7-AAD double labeling. Cells undergoing early apoptosis are positive for annexin V and negative for 7-AAD, necrotic cells are 7-AAD positive and annexin V negative, and late apoptotic cells are positive for annexin V and 7-AAD staining. As shown in Figure 3A, the proportions of early and late apoptotic cells in HEC-1-A and RL95-2 were significantly increased after 2 h of tramadol treatment and also increased in a time-dependent manner. 

**Figure 4:**
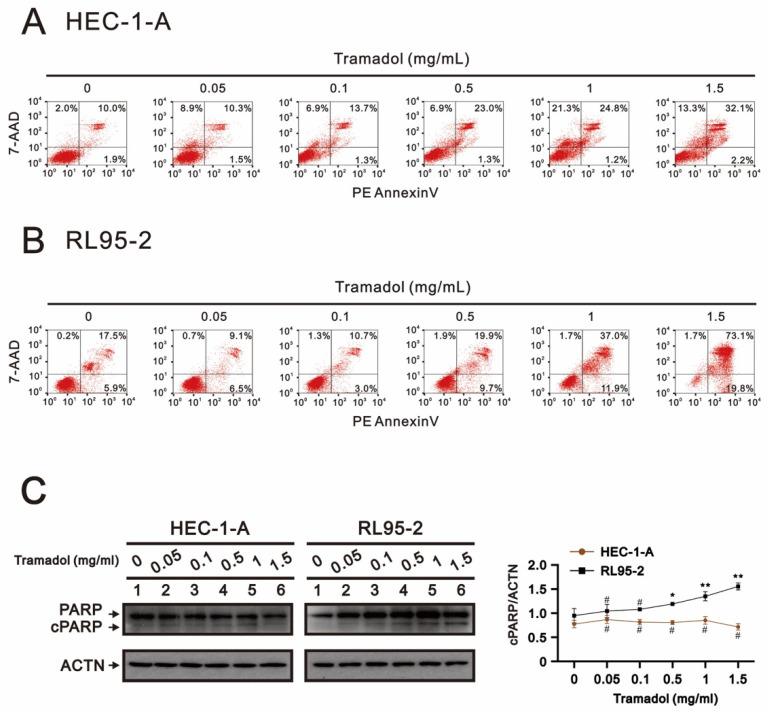
Effects of tramadol on apoptosis of human endometrial cancer cells. (**A**,**B**) HEC-1-A and RL95-2 cells were treated with tramadol (0, 0.05, 0.1, 0.5, 1, and 1.5 mg/mL) for 24 h. (**C**) HEC-1-A and RL95-2 cells were treated with tramadol (0, 0.05, 0.1, 0.5, 1, and 1.5 mg/mL) for 4 h. Cell lysates were subjected to Western blot analysis using antibodies against cPARP. ACTN was the protein loading control. The protein bands were quantified through pixel density scanning and evaluated using ImageJ, version 1.44a (http://imagej.nih.gov/ij/) (accessed on 12 December 2022). The ratios of cPARP/ACTN were plotted. Bars depict the mean ± SD of three independent experiments. Student’s *t*-tests were performed, and the results were compared with the vehicle. ^#^ *p* > 0.05, * *p* < 0.05, and ** *p* < 0.01 (Student’s *t*-test).

In Figure 4A, tramadol increased the number of late apoptotic (from 10% to 32.1%) and necrotic cells (from 2% to 13.3%) in HEC-1-A cells in a dose-dependent manner. In RL95-2 cells, tramadol increased the early apoptotic (from 5.9% to 19.8%) and late apoptotic (from 17.5% to 73.1%) cell populations, but did not increase the necrotic cell population in Figure 4B. In addition, we also investigated whether a well-known apoptotic biomarker, cleavage of poly(ADP-ribose) polymerase (cPARP), was altered by tramadol in HEC-1-A and RL95-2 cells. The Western blotting results showed that tramadol significantly increased the amounts of cPARP protein in RL95-2 cells, but not in HEC-1-A cells (Figure 4C). These results suggest that tramadol may induce different cell death types in HEC-1-A and RL95-2 cells.

### 2.3. Tramadol Caused Different Types of Cell Death in Two Types of Human Endometrial Cancer Cells

**Figure 5:**
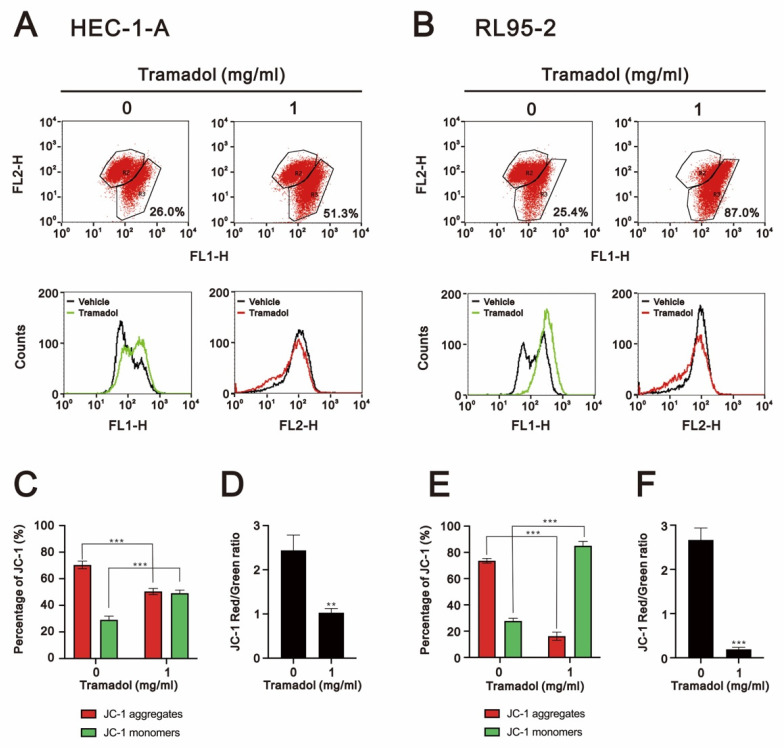
Effects of tramadol on the mitochondrial membrane potential of human endometrial cancer cells. (**A**,**B**) HEC-1-A and RL95-2 cells were treated with tramadol (0 and 1 mg/mL) for 24 h. Mitochondrial membrane potential was assessed using JC-1 staining with flow cytometry. (**C**,**E**) The percentages of red and green fluorescences were plotted. (**D**,**F**) The red/green fluorescence intensity ratios were measured and plotted. Bars depict the mean ± SD of three independent experiments. Student’s *t*-tests were performed, and the results were compared with the vehicle. ** *p* <0.01 and *** *p* < 0.001 (Student’s *t*-test).

Since mitochondrial depolarization is an irreversible change during apoptosis, the phenotype of early apoptosis is known to be associated with plasma membrane phosphatidylserine externalization and is often accompanied by loss of mitochondrial membrane potential [19]. We then examined the effect of tramadol on mitochondrial membrane potential in HEC-1-A and RL95-2 cells using JC-1 and flow cytometry. In non-apoptotic cells, JC-1 aggregates in dimer form in the mitochondria and emits red fluorescence. In apoptotic cells, JC-1 exists as a monomer in the cytoplasm and emits green fluorescence. Thus, mitochondrial depolarization could be measured as a function of a decrease in the red/green fluorescence intensity ratio. Tramadol significantly increased the loss of mitochondrial membrane potential from 26% to 51.3% in HEC-1-A cells (Figure 5A,C green bars) and from 25.4% to 87% in RL95-2 cells (Figure 5B,E green bars). We further measured the ratios of JC-1 red/green, and these ratios were significantly reduced in both types of endometrial cancer cells (Figure 5D,F), suggesting that mitochondrial depolarization was induced by tramadol. 

**Figure 6:**
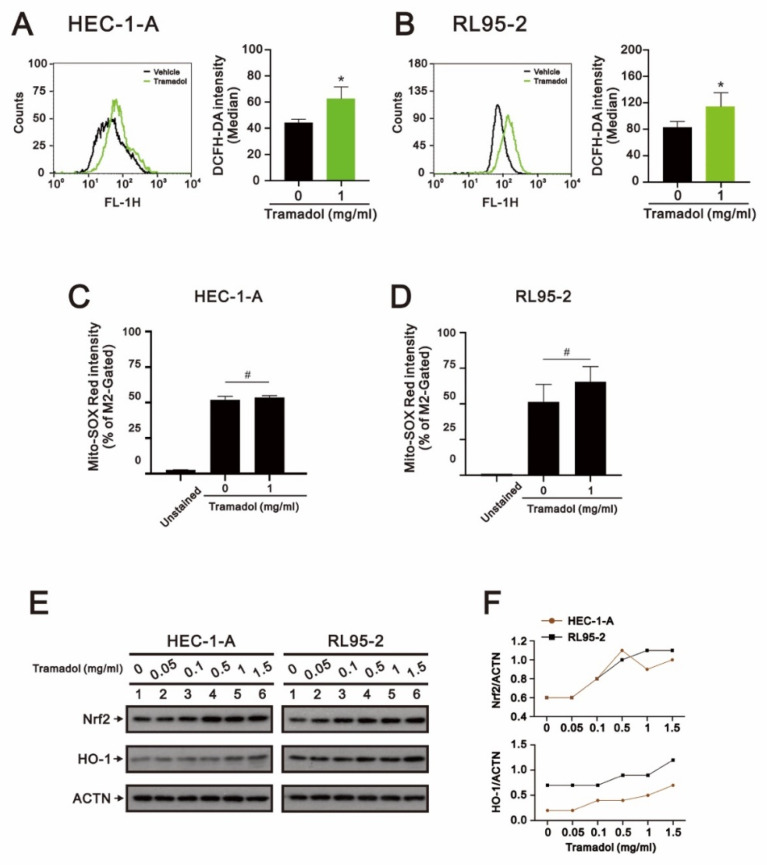
Effects of tramadol on ROS levels of human endometrial cancer cells. (**A**–**D**) HEC-1-A and RL95-2 cells were treated with tramadol (0 and 1 mg/mL) for 2 h. Intracellular ROS and mtROS levels were assessed using DCFH-DA and MitoSOX Red staining with flow cytometry. Bars depict the mean ± SD of three independent experiments. Student’s *t*-tests were performed, and the results compared with the vehicle. ^#^ *p* > 0.05 and * *p* < 0.05 (Student’s *t*-test). (**E**) HEC-1-A and RL95-2 cells were treated with tramadol (0, 0.05, 0.1, 0.5, 1, and 1.5 mg/mL) for 4 h. Cell lysates were subjected to Western blot analysis using antibodies against the indicated proteins. ACTN was the protein loading control. (**F**) The protein bands from (**E**) were quantified through pixel density scanning and evaluated using ImageJ, version 1.44a (http://imagej.nih.gov/ij/) (accessed on 12 December 2022). The ratios of protein/ACTN, including Nrf2 and HO-1, were plotted.

Most cancer cells have been found to frequently express increased levels of antioxidant proteins that are highly adaptable to elevated levels of ROS [20]. However, once ROS levels are raised above the cytotoxic threshold of cancer cells by triggering ROS accumulation or inhibition of the ROS scavenging systems, ROS-induced oxidative stress can lead to apoptosis [21]. We then investigated whether tramadol causes cytotoxicity by increasing ROS levels in HEC-1-A and RL95-2 cells. After tramadol treatment, the levels of intracellular ROS and mtROS in HEC-1-A and RL95-2 cells were detected using DCFH-DA and Mito-SOX Red with flow cytometry, respectively. The results in Figure 6A,B show that the levels of intracellular ROS were significantly increased in HEC-1-A and RL95-2 cells, while the mtROS levels were not significantly changed, as shown in Figure 6C,D. NF-E2-related factor 2 (Nrf2) is an important transcription factor that controls gene expression of endogenous antioxidant synthesis and ROS-eliminating enzymes [22]. When cellular ROS levels are excessive, Nrf2 induces expression of cytoprotective genes such as HO-1 in response to extensive oxidative stress [23]. Therefore, we examined the protein expression of Nrf2 and HO-1 in tramadol-treated endometrial cancer cells using Western blotting. The results in Figure 6E,F showed that tramadol increased Nrf2 and HO-1 protein expression in HEC-1-A and RL95-2 cells, which is consistent with the results shown in Figure 6A,B.

**Figure 7:**
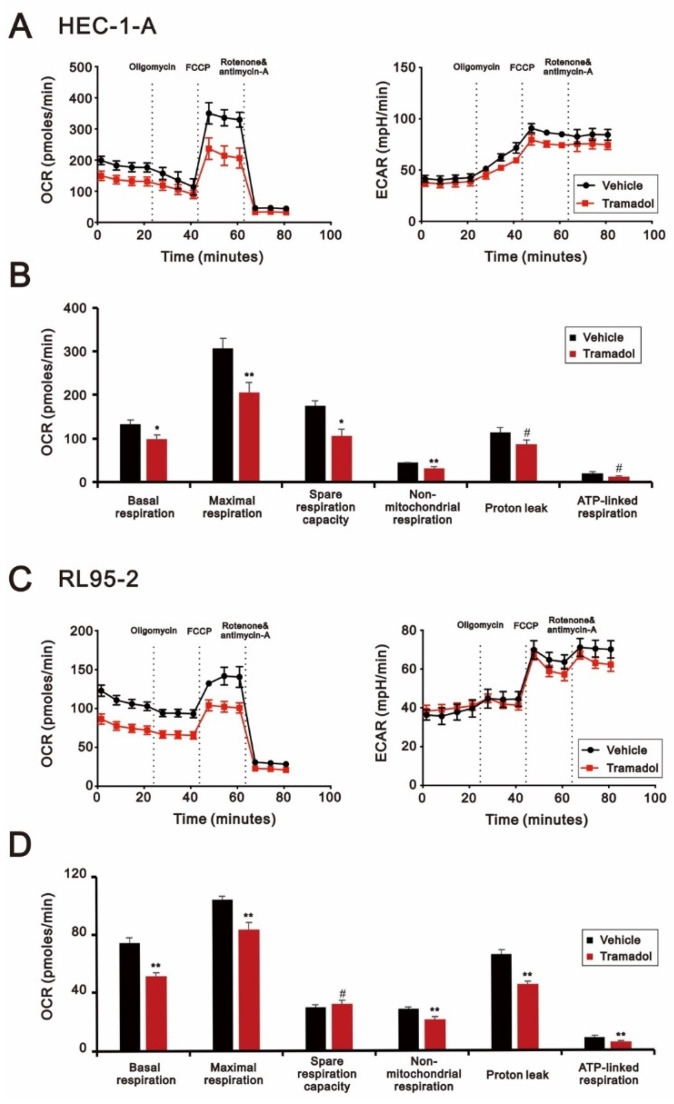
Effects of tramadol on mitochondrial function of human endometrial cancer cells. (**A**,**B**) HEC-1-A and (**C**,**D**) RL95-2 cells were treated with tramadol (0 and 1 mg/mL) for 4 h. (**A**–**D**) OCR and (**A**,**C**) ECAR were assessed using seahorse XFp analyzers. Bars depict the mean ± SD of three independent experiments. Student’s *t*-tests were performed, and the results were compared with the vehicle. ^#^ *p* > 0.05, * *p* < 0.05, ** *p* < 0.01 (Student’s *t*-test).

Given that tramadol induced apoptosis and loss of mitochondrial membrane potential in HEC-1-A and RL95-2 cells (Figure 3, Figure 4A,B and Figure 6A,B), we expected that tramadol might affect mitochondrial function in human endometrial cancer cells. To assess the changes in mitochondrial function, we measured the oxygen consumption rate (OCR), extracellular acidification rate (ECAR), and mitochondrial DNA (mtDNA) copy number of HEC-1-A and RL95-2 cells after tramadol treatment. OCR and ECAR values have been identified as indicators of mitochondrial respiration and glycolysis [24]. In Figure 7A,C, the results show that tramadol significantly reduced OCR in HEC-1-A and RL95-2 cells but had no significant effect on ECAR. In HEC-1-A and RL95-2 cells, tramadol treatment significantly reduced mitochondrial function, including basal, maximal, and spare respiration capacity (Figure 7B,D). Tramadol also significantly reduced proton leak and ATP-linked respiration in RL95-2 cells (Figure 7D) and tended to reduce proton leak and ATP-linked respiration in HEC-1-A cells (Figure 7B). This result suggests that tramadol affects oxidative phosphorylation, but not glycolysis, in endometrial cancer cells.

**Figure 8:**
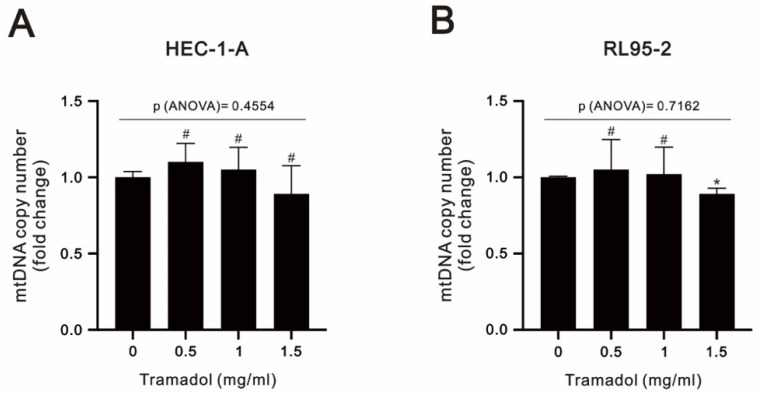
Effects of tramadol on the mtDNA copy number of human endometrial cancer cells. (**A**,**B**) HEC-1-A and RL95-2 cells were treated with tramadol (0, 0.5, 1, and 1.5 mg/mL) for 24 h. Bars depict the mean ± SD of three independent experiments. Student’s *t*-tests were performed, and the results were compared with the vehicle. ^#^ *p* > 0.05 and * *p* < 0.05 (Student’s *t*-test). (**A**,**B**) The means between the groups were analyzed by one-way ANOVA.

The copy number of mtDNA is an important component and indicator of overall mitochondrial health [25]. In Figure 8A,B, our analytic results show that there were no evident changes caused by tramadol in mtDNA content by the analysis of one-way ANOVA, suggesting that mitochondrial mass was maintained. 

### 2.4. Tramadol Acted Synergistically with Doxorubicin and Cisplatin in Human Endometrial Cancer Cells

**Figure 9:**
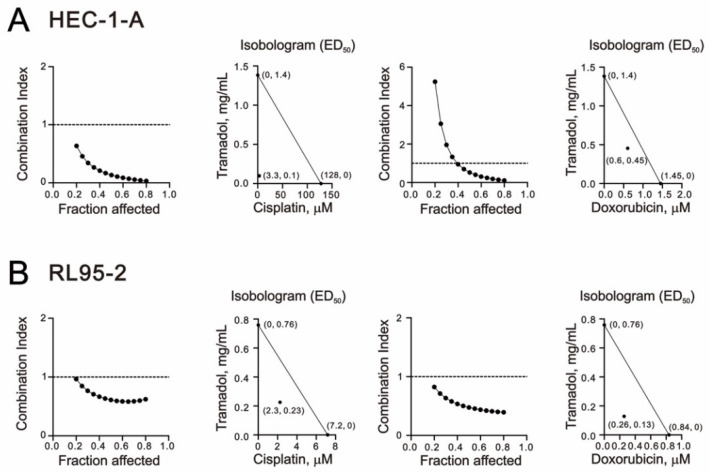
Combination index (CI) of tramadol in combination with cisplatin and doxorubicin in human endometrial cancer cells. (**A**) HEC-1-A cells were treated with tramadol (0, 0.005859, 0.011719, 0.023438, 0.046875, 0.09375, 0.1875, 0.375, 0.75, and 1.5 mg/mL), cisplatin (0, 1.5625, 3.125, 6.25, 12.5, 25, 50, and 100 μM), and doxorubicin (0, 0.0625, 0.125, 0.25, 0.5, 1, 2, and 4 μM) for 24 h. (**B**) RL95-2 cells were treated with tramadol (0, 0.003906, 0.007813, 0.015625, 0.03125, 0.0625, 0.125, 0.25, 0.5, and 1 mg/mL), cisplatin (0, 0.3125, 0.625, 1.25, 2.5, 5, 10, and 20 μM), and doxorubicin (0, 0.0625, 0.125, 0.25, 0.5, 1, 2, and 4 μM) for 24 h.

In clinical applications, the importance of combination chemotherapy has been suggested as a means to achieve synergistic effects and minimize drug doses for cancer treatment [26]. Here, we used a combination index to examine the interaction of the two drugs in HEC-1-A and RL95-2 cells by combining tramadol with cisplatin and doxorubicin, commonly used chemotherapy drugs in patients with endometrial cancer. As shown in Figure 9A,B, we found that tramadol had synergetic effects with cisplatin and doxorubicin in HEC-1-A and RL95-2 cells. When tramadol was used in combination with cisplatin or doxorubicin, the ED_50_ of cisplatin decreased from 128 to 3.3 μM in HEC-1-A cells and from 7.2 to 2.3 μM in RL95-2 cells, and the ED_50_ of doxorubicin decreased from 1.45 to 0.6 μM in HEC-1-A cells and 0.84 to 0.26 μM in RL95-2 cells.

**Figure 10:**
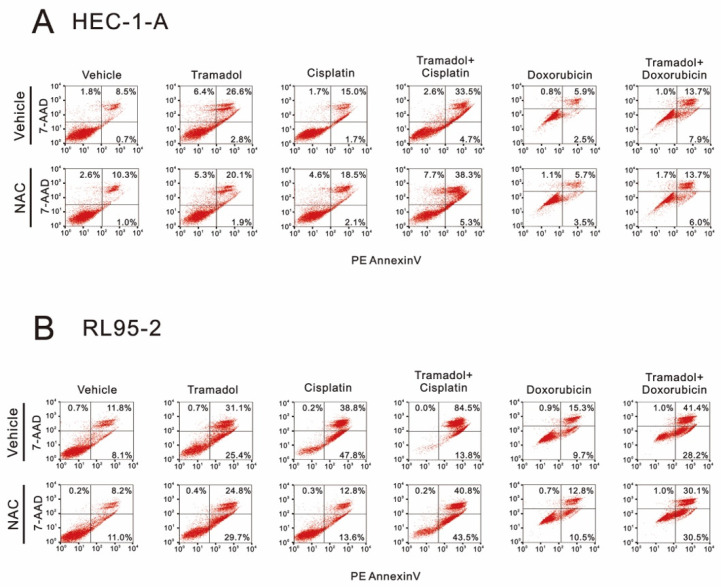
Effects of NAC pretreatment on tramadol-induced apoptosis of human endometrial cancer cells. (**A**) The vehicle groups of HEC-1-A cells were treated with 1 mg/mL tramadol, 128 μM cisplatin, and 1.45 μM doxorubicin for 24 h. The NAC groups were pre-treated with 5 mM NAC for 2 h. (**B**) The vehicle groups of RL95-2 cells were treated with 1 mg/mL tramadol, 7.2 μM cisplatin, and 0.84 μM doxorubicin for 24 h. The NAC groups were pre-treated with 5 mM NAC for 2 h.

To determine whether tramadol combined with doxorubicin and cisplatin enhances apoptosis in HEC-1-A and RL95-2 cells, we used annexin V and 7-AAD and flow cytometry to detect apoptosis following combination therapy. In addition, we also tested whether tramadol induces apoptosis by increasing intracellular ROS through pretreatment with N-acetyl cysteine (NAC). In HEC-1-A cells, tramadol increased cisplatin-induced late apoptosis from 15% to 33.5% and doxorubicin-induced late apoptosis from 5.9% to 13.7% (Figure 10A). Furthermore, tramadol-induced apoptosis was inhibited after NAC pretreatment, but not in the combined treatment group (Figure 10A). In RL95-2 cells, tramadol increased cisplatin-induced late apoptosis from 38.8% to 84.5% and doxorubicin-induced late apoptosis from 15.3% to 41.4% (Figure 10B). However, after NAC pretreatment, tramadol-induced apoptosis showed a trend of switching from late to early apoptosis, either treated alone or in combination with cisplatin and doxorubicin (Figure 10B).

## 3. Discussion

In the treatment of endometrial cancers, tramadol is commonly used to alleviate patients’ postoperative pain or cancer pain. Recently, studies have indicated that tramadol may have anticancer effects by upregulating cell apoptosis, promoting oxidative stress, and modulating natural killer cell activity [27,28,29,30]. Our previous work also suggested an antitumor effect of tramadol on breast cancers [11]. Considering that endometrial cancer is the most common gynecological malignancy, its incidence and mortality have continued to rise in recent years. Most patients with early-stage endometrial cancer can be cured with surgery alone or in combination with adjuvant pelvic radiation, but patients with metastatic or recurrent endometrial cancer have very limited response rates to cytotoxic chemotherapy, targeted agents, or hormonal therapy [31]. Here, we investigated the cytotoxic mechanism of tramadol in human endometrial cancer cells. The results of our study suggested that tramadol may have antitumor effects in human endometrial cancer. When RL95-2 (type I) and HEC-1-A (type II) human endometrial cancer cells were treated with tramadol, we found that tramadol induced apoptosis, cell cycle arrest, and increased intracellular ROS levels, the inhibition of colony formation and the cell proliferative capacity of two human endometrial cancer cells. We also found that RL95-2 cells were more sensitive to tramadol than HEC-1-A cells. 

The tramadol concentrations currently used in our in vitro study should be higher than those used in clinical practice, because tramadol exerts an analgesic effect in the human body through the metabolism of cytochrome P450 (CYP) 2D6 into active metabolites [32]. Its metabolites have approximately 700-fold higher affinity for μ-receptors than tramadol [33]. In addition, our current dose is similar to that studied by Kim and colleagues [10]. They also conducted further study in an animal model and concluded that clinical doses of tramadol (1.5–3.0 mg kg^−1^) attenuate the growth of MCF-7 cell-derived breast cancer in xenograft mice [34]. Since tramadol is widely used clinically, the safety of tramadol has been confirmed in previous drug clinical trials. Additionally, tramadol has not been shown to be carcinogenic in clinical and animal studies.

Endometrial cancer usually affects postmenopausal women who are commonly associated with comorbidities. Increasing comorbidity is related to poorer survival among all endometrial cancer patients [35]. Chemotherapy should be more precise so as to avoid severe complications, especially for those who have type II endometrial cancer requiring prolonged salvage chemotherapy for cancer recurrence. Unfortunately, thus far, there are no approved target therapies available for endometrial cancer. Enhancing current mainstay chemotherapy agents would be an alternative. In the present study, the synergistic effects of tramadol in combination with cisplatin and doxorubicin were observed in endometrial cancer cell lines, and these combinations were also found to enhance cisplatin- and doxorubicin-induced apoptosis. A high level of ROS production has been shown to induce autophagy, apoptosis, and necrosis in cells [16]. Interestingly, when we attempted to rescue tramadol-induced apoptosis using an ROS scavenger, NAC, we found that tramadol-induced apoptosis was rescued in HEC-1-A cells, but not when we used tramadol combined with cisplatin and doxorubicin. Meanwhile, in RL95-2 cells, NAC appeared to alter the apoptosis induced by tramadol and tramadol in combination with cisplatin and doxorubicin from late to early apoptosis, but still failed to rescue apoptosis. These results suggest that intracellular ROS induced by tramadol may play different roles in the two types of endometrial cancer cells. Apoptosis is a form of programmed cell death. It differs from necrosis, which is the unprogrammed death of cells due to damage [36]. To develop effective treatments for endometrial cancer, it is necessary to explore the differences in the mechanisms of cell death induced by tramadol in the two types of endometrial cancer cells.

Traditional clinical endometrial cancer treatments, such as surgery, chemotherapy, and radiation therapy have limited efficacy due to drug resistance and side effects. There is growing evidence that selectively delivering drugs to specific subcellular organelles can significantly improve the efficiency of cancer treatment [37]. Due to the role of mitochondria in regulating the apoptosis and metabolism of cancer cells, mitochondrial-targeted therapeutic strategies are expected to be used in cancer therapy [38,39]. We also focused on the effect of tramadol on mitochondria in human endometrial cancer cells, which plays an important role in activating apoptosis. Tramadol was shown to cause a decrease in mitochondrial membrane potential and inhibited mitochondrial respiratory function in human endometrial cancer cells. It has previously been reported that renal injury associated with high-dose or chronic tramadol administration is caused by mitochondrial dysfunction, including mitochondrial depolarization, ATP depletion, mitochondrial permeabilization, lipid peroxidation, and decreased mitochondrial dehydrogenase activity [40]. Chronic tramadol treatment also induces memory impairment by inducing brain mitochondrial dysfunction [41]. The cited works all demonstrate the importance of mitochondrial damage in the mechanism of tramadol-induced cytotoxicity, which was also confirmed in our study of endometrial cancer cells. The Warburg effect suggests that glycolysis is upregulated in cancer cells compared to normal cells, leading many to hypothesize that OXPHOS is downregulated in all cancers. However, recent studies have indicated that, even in the presence of active glycolysis, OXPHOS can also be upregulated in certain cancers, including leukemia, lymphoma, and endometrial cancer [42,43]. Furthermore, serous endometrial carcinomas have been shown to have significantly higher mtDNA copy numbers than all other subtypes [44]. This characteristic of OXPHOS upregulation in endometrial cancers may potentially sensitize them to OXPHOS inhibition by tramadol treatment, resulting in cytotoxicity. 

## 4. Materials and Methods

### 4.1. Cell Culture and Reagents

Human endometrial adenocarcinoma cell lines RL95-2 (ATCC^®^CRL-1671™) and HEC-1-A cells (ATCC^®^HTB-112™) were obtained from the American Type Culture Collection (ATCC; Manassas, VA, USA). For the cell culture experiments, we cultured RL95-2 and HEC-1-A cells in Dulbecco’s Modified Eagle Medium Nutrient Mixture F-12 (DMEM/F12) and McCoy’s 5A medium supplemented with 10% fetal bovine serum (FBS) and 1% penicillin–streptomycin (Thermo Fisher Scientific, Waltham, MA, USA), respectively. Tramadol, doxorubicin, cisplatin, 2′,7-dichlorofluorescein diacetate (DCFH-DA), propidium iodide (PI), and thiazolyl blue tetrazolium bromide (MTT) were obtained from Sigma Aldrich (Sigma Aldrich; St. Louis, MO, USA). MitoSOX Red was obtained from Invitrogen (Invitrogen; Waltham, MA, USA). 

### 4.2. Colony Formation Assay

Cells were seeded into 6-well cell culture plates (2000 cells per well) and incubated for 14 days. The cells were then stained with 0.005% crystal violet solution for 1 h. After air drying, colonies were photographed and counted. 

### 4.3. Cell Proliferation Assay

Cell proliferation was assessed using the FITC BrdU flow kit (BD Biosciences). After being treated with tramadol, the cells were labeled with 10 mM BrdU for 1 h. Then, the cells were stained with FITC-conjugated anti-BrdU according to the manufacturer’s instructions. Each sample was measured using a FACSCalibur flow cytometer and Cell Quest Pro software.

### 4.4. Cell Cycle Profiles

Cell cycle profiles were assessed by measuring cellular DNA content using propidium iodide (PI) staining. After being treated with the drugs, the treated cells were trypsinized and washed with PBS. We resuspended the cell pellet in 1 mL PBS and fixed them in 5 mL 70% ice-cold ethanol, then stored them at −30 °C overnight. On the following day, the cells were washed twice with ice-cold PBS containing 1% FBS and centrifuged at 4 °C with 1000 rpm for 5 min. Then, they were stained with a PI staining solution (5 μg/mL PI in PBS, 0.5% Triton X-100, and 0.5 μg/mL RNase A) for 30 min at 37 °C in the dark. Each sample was measured using the FACSCalibur flow cytometer and Cell Quest Pro software. 

### 4.5. Apoptosis Assay

Apoptosis was assessed using the PE Annexin-V apoptosis detection kit (BD Pharmingen, San Diego, CA, USA) with 7-AAD according to the manufacturer’s protocol. The apoptotic ratio was measured using the FACSCalibur flow cytometer and Cell Quest Pro software (BD Biosciences, Franklin Lakes, NJ, USA).

### 4.6. Mitochondrial Membrane Potential Assay

Mitochondrial membrane potential was monitored using the MitoScreen (JC-1) kit (BD Pharmingen). After being treated with tramadol, dead and live cells were collected, and JC-1 solution was added prior to the 15 min incubation. The cells were then washed twice with a binding buffer. Each sample was evaluated using the FACSCalibur flow cytometer and Cell Quest Pro software.

### 4.7. Reactive Oxygen Species (ROS) Assay

We performed the detection of intracellular ROS and mitochondrial ROS levels using DCFH-DA and MitoSOX Red staining, respectively. After being treated with tramadol, the treated cells were washed with PBS twice and incubated with 10 μM DCFH-DA or 5 μM MitoSOX Red at 37 °C for 30 min in the dark. We washed the treated cells with PBS and evaluated them using the FACSCalibur flow cytometer and Cell Quest Pro software.

### 4.8. Western Blot

Following the tramadol treatment, the cells were lysed in an RIPA (radio-immunoprecipitation assay) cell lysis buffer for protein extraction. Using the DC protein assay, equal amounts of protein were determined for SDS-PAGE. Then, the proteins from the SDS-PAGE gel were transferred to PVDF membranes, which was then blocked with 5% nonfat milk with TBST for 1 h. The proteins were incubated with primary antibodies overnight at 4 °C with shaking and then incubated with secondary antibodies for an additional 1 h at room temperature. The primary antibodies of cPARP were obtained from Cell Signaling Technology (Danvers, MA, USA); Nrf2 and ACTN were obtained from Santa Cruz Biotechnology (Santa Cruz, CA, USA); HO-1 was obtained from Enzo Life Sciences (Farmingdale, NY, USA).

### 4.9. Detection of the Oxygen Consumption Rate (OCR) and Extracellular Acidification Rate (ECAR)

The cellular OCR and ECAR were evaluated using a Seahorse XFp Analyzer according to the manufacturer’s instructions (Agilent, Santa Clara, CA, USA). Briefly, after the tramadol treatment, we replaced the used medium with sodium bicarbonate-free DMEM pH 7.4 supplemented with 2% FBS and 2% horse serum for 1 h. The OCR and ECAR of the cells were measured sequentially before and after the addition of oligomycin (1 μM), FCCP (0.5 μM), and rotenone/antimycin-A (0.5 μM).

### 4.10. Measurement of Mitochondrial DNA (mtDNA) Copy Number

The quantification of mtDNA copy number was assessed by the Absolute Human Mitochondrial DNA Copy Number Quantification qPCR Assay Kit. (ScienCell Research Laboratories, San Diego, CA, USA) according to the manufacturer’s protocol. Briefly, after the tramadol treatment, genomic DNA extraction from cells using the gSYNC^TM^ DNA Extraction Kit (Geneaid, Taipei, Taiwan). For qPCR reaction (per sample): 1 μL of DNA template (5 ng/μL), 2 μL of mtDNA or single copy reference (SCR) primer stock solution, 10 μL of 2X GoldNStart TaqGreen qPCR master mix, and 7 μL of nuclease-free H_2_O. For qPCR program setup: Initial denaturation at 95 °C for 10 min, followed by 32 cycles with denaturation at 95 °C for 20 s, annealing at 52 °C for 20 s, and extension at 72 °C for 45 s. A reference human genomic DNA sample with a known concentration (mtDNA of 1.27 ± 0.03 × 10^3^ copies per diploid cell) was used.

### 4.11. Cell Survival Analysis

Cells were seeded into 96-well plates, incubated for 24 h, and then treated with different concentrations of tramadol, cisplatin, and doxorubicin for 24 h. We added 0.5 mg/mL MTT solution to each well and incubated them for 2 h. We removed the MTT solution and added dimethyl sulfoxide (DMSO; 100 μL). The absorbances at 570 nm and 650 nm were measured using an ELISA plate reader (Multiskan EX, Thermo Fisher Scientific, Waltham, MA, USA). The combination index (CI) was calculated using CalcuSyn (Biosoft, Cambridge, UK) to generate an isobologram. CI values of 1, <1, and >1 indicate additive, synergistic, and antagonistic effects, respectively.

### 4.12. Statistical Analysis

Values were expressed in mean ± SD, and the data are representative of three independent experiments. All the comparisons between groups were performed using Student’s *t*-tests. Comparison among multiple groups was conducted using analysis of variance (ANOVA). Statistical significance was set at *p* < 0.05.

## 5. Conclusions

In this study, we investigated the cytotoxicity mechanism of tramadol on endometrial cancer cells. Our findings demonstrate that tramadol significantly inhibits the proliferation of endometrial cancer cells, induces ROS production, leads to mitochondrial dysfunction, and ultimately leads to apoptosis. Furthermore, tramadol has synergistic effects with cisplatin and doxorubicin in endometrial cancer cells. These findings provide a potential adjuvant therapy strategy for the treatment of endometrial cancer.

## Data Availability

Not applicable.
